# Understanding the Remodelling of Cell Walls during *Brachypodium distachyon* Grain Development through a Sub-Cellular Quantitative Proteomic Approach

**DOI:** 10.3390/proteomes4030021

**Published:** 2016-06-24

**Authors:** Mathilde Francin-Allami, Virginie Lollier, Marija Pavlovic, Hélène San Clemente, Hélène Rogniaux, Elisabeth Jamet, Fabienne Guillon, Colette Larré

**Affiliations:** 1 UR1268 BIA (Biopolymères Interactions Assemblages), INRA, Nantes 44300, France; virginie.lollier@nantes.inra.fr (V.L.); marapavlovic86@gmail.com (M.P.); helene.rogniaux@nantes.inra.fr (H.R.); Fabienne.Guillon@nantes.inra.fr (F.G.); colette.larre@nantes.inra.fr (C.L.); 2 Laboratoire de Recherche en Sciences Végétales, Université de Toulouse, CNRS, UPS, 24 Chemin de Borderouge-Auzeville, BP42617, Castanet-Tolosan 31326, France; sancle@lrsv.ups-tlse.fr (H.S.C.); jamet@lrsv.ups-tlse.fr (E.J.)

**Keywords:** *Brachypodium distachyon*, cell wall, cell wall proteins, grain development, grass, quantitative mass spectrometry, *Pooideae*, polysaccharide remodelling, proteomics

## Abstract

*Brachypodium* *distachyon* is a suitable plant model for studying temperate cereal crops, such as wheat, barley or rice, and helpful in the study of the grain cell wall. Indeed, the most abundant hemicelluloses that are in the *B. distachyon* cell wall of grain are (1-3)(1-4)-β-glucans and arabinoxylans, in a ratio similar to those of cereals such as barley or oat. Conversely, these cell walls contain few pectins and xyloglucans. Cell walls play an important role in grain physiology. The modifications of cell wall polysaccharides that occur during grain development and filling are key in the determination of the size and weight of the cereal grains. The mechanisms required for cell wall assembly and remodelling are poorly understood, especially in cereals. To provide a better understanding of these processes, we purified the cell wall at three developmental stages of the *B. distachyon* grain. The proteins were then extracted, and a quantitative and comparative LC-MS/MS analysis was performed to investigate the protein profile changes during grain development. Over 466 cell wall proteins (CWPs) were identified and classified according to their predicted functions. This work highlights the different proteome profiles that we could relate to the main phases of grain development and to the reorganization of cell wall polysaccharides that occurs during these different developmental stages. These results provide a good springboard to pursue functional validation to better understand the role of CWPs in the assembly and remodelling of the grain cell wall of cereals.

## 1. Introduction

For less than fifteen years, *Brachypodium distachyon* has stood out as the plant model for the structural and functional genomics of temperate grasses [1,2]. It has a small genome and a relatively short lifecycle, and it is self-pollinating and genetically transformable. It belongs to the *Pooideae* subfamily of the grasses (*Poaceae*), which includes the cereals *Triticum aestivum* (wheat), *Hordeum vulgare* (barley) and *Avena sativa* (oat). The genome of *B. distachyon* was the first *Pooideae* genome to be sequenced [3].

The structure and development of the *B. distachyon* grain is well documented for the sequenced Bd21 accession [4,5,6,7,8]. Grain development is broadly similar between cereals and *B. distachyon*, although there are a number of significant differences in the cellular differentiation and gene expression patterns. The *B. distachyon* grain presents a prominent and persistent nucellar epidermis and a thick wall in the endosperm, like that of rice. The aleurone forms a continuous layer of living cells, even at grain maturity. It remains attached to the endosperm and is less differentiated from the rest of endosperm than in wheat [8].

The composition of the *B. distachyon* grain and of its endosperm is well characterised. It contains a high percentage of proteins (17% of dry matter in mature grain [4]), with a predominance of globulins represented mainly by glutelins similar to those of rice or oat. Prolamins, which are the major wheat storage proteins, were also detected in the *B. distachyon* grain but to a lesser extent [6]. Starch is detectable in the *B. distachyon* grain at approximately 13 days after flowering (DAF) and only accounts for 10% of its final dry matter. In other cereal crops, this polymer reaches 50%–70% of the dry matter, thus representing the main carbohydrate storage molecule. Furthermore, the *B. distachyon* grain has been shown to have thick cell walls, particularly in the endosperm, that are highly enriched in β-(1-3)(1-4) glycans, also called mixed-linked β-glucan. It was proposed that this polysaccharide could serve as the storage carbohydrate of the *B. distachyon* grain that could be mobilised during germination [4,8]. Mixed-linked β-glucan appears in the endosperm walls at the cellularisation stage of development. At first, it may act as a structural compound, but then, it rapidly accumulates to become the predominant polysaccharide in the mature grain. The thick cell walls of the endosperm make it a hard grain, allowing the cell walls to act as a physical barrier contributing to seed defence in addition to their function as storage. Additionally, the *B. distachyon* grain contains significant amounts of cellulose and arabinoxylans (AXs). AXs are highly feruloylated and contain a high proportion of arabinose disubstitution compared to other cereals [4,9]. Altogether, the *B. distachyon* grain has a cell wall polysaccharide profile similar to those of barley and oat because it has a higher amount of mixed-linked β-glucan than AXs. Like main grasses, *B. distachyon* cell walls contain very low amounts of mannan and pectin, which could, however, play important roles in the assembly of cell walls and influence their physico-chemical characteristics [10].

During grain development, cell wall metabolism is probably the result of simultaneous synthesis/assembly and degradation/remodelling events. Several gene families were identified to be involved in cell wall biosynthesis [11,12]. While synthesis/assembly is assumed to be more intensive during the early stage of development, degradation and remodelling are thought to be predominant in the later steps. However, the remodelling of cell walls occurs during development, leading to increases in polysaccharide lengths, variation in the substitution degree of polysaccharides, cross-linking between cell wall constituents, variation in methylation and acetylation degree of polysaccharides [10].

The remodelling of cell wall polysaccharides during plant development involves numerous proteins and enzymes. Many cell wall proteins (CWPs) have been identified by specific proteomic approaches (for recent reviews, see [13,14]). Regarding *B. distachyon*, several studies have been performed on young seedlings, leaves, stems and grain [15,16,17]. Among the identified CWPs, the glycoside hydrolases (GHs) are known to play a major role, with approximately twenty GH families potentially involved in cell wall remodelling, with glycosidase, glucanase and transglycosidase activities [18,19]. These GH families are mainly characterised for dicot plants [18]. As an example, xyloglucan endotransglucosylase/hydrolases (XTHs) belonging to the GH16 family were shown to play a role in the variation of xyloglucan polymer sizes in tobacco leaves or *Arabidopsis thaliana* roots [20,21]. Other CWPs were also demonstrated to participate in cell wall remodelling, such as carbohydrate esterases (CEs), carbohydrate lyases (CLs), class III peroxidases (Prxs) and expansins [18,22]. The CE family includes pectin methylesterases (PMEs) that are known to be important in regulating cell expansion in plants [23].

Knowledge concerning the expression and function of these CWPs during grain development is lacking, especially in monocot plants. In a previous study, we investigated the cell wall proteome of the *B. distachyon* grain at a single developmental stage, i.e., the beginning of storage accumulation, and we highlighted numerous CWPs possibly involved in cell wall remodelling [16]. To increase the understanding of this process, we investigated the CWPs of the *B. distachyon* grain at three distinct developmental stages, starting at the end of cellularisation and ending at the beginning of the differentiation stage. A comparative analysis of the identified CWPs and a label-free quantitative analysis of the CWPs common to the three developmental stages were performed. The relationships between the observed changes in the proteome profiles and the evolution of the grain polysaccharide composition were assessed.

## 2. Experimental Section

### 2.1. Plant Material

*B. distachyon* accession Bd21 was grown at 24 °C day/18 °C night with a photoperiod of 20 h light/4 h dark. The grains were harvested at 9, 13 or 19 days after flowering (DAF) and frozen in liquid nitrogen prior to storage at −20 °C.

### 2.2. Cell Wall Purification and Protein Extraction

Cell wall purification was performed as described [16] following a protocol adapted from [24]. The proteins were extracted from the purified cell walls using 0.2 M CaCl_2_ (twice) and 2 M LiCl (twice) solutions as described [25]. Five biological replicates were performed per developmental stage.

### 2.3. Mass Spectrometry Analysis

The cell wall proteins (50 μg) were separated by 1D-electrophoresis (1D-E) in 12% polyacrylamide gels. For each biological replicate, the corresponding gel lane was excised from the gel and cut into twelve slices of approximately 1.7 mm in width. The proteins were further reduced, alkylated and trypsinolysed, as previously described, prior to mass spectrometry (MS) analyses [6].

Nanoscale LC-MS/MS analyses of the samples were performed using an Ultimate 3000 RSLC system (Thermo-Fisher Scientific, Waltham, MA, USA) coupled with a LTQ-Orbitrap VELOS mass spectrometer (Thermo-Fisher Scientific). Chromatographic separation was conducted on a reverse-phase capillary column (AcclaimPepmap C18 2 μm, 100 Å, 75 μm id × 15 cm in length, Thermo-Fisher Scientific) at a flow rate of 300 nL/min, as previously described [26]. The MS data acquisitions were performed using the Xcalibur 2.1 software. Full MS scans were acquired at a high resolution (FWMH 30,000) using an Orbitrap analyser (*m*/*z*: 400–2000), while the CID spectra were recorded for the five most intense ions in the linear LTQ traps. The LC-MS/MS spectrum files corresponding to the 180 samples were processed via the X!Tandem pipeline, available at http://pappso.inra.fr/bioinfo/xtandempipeline, with the X!Tandem version TORNADO. Some replicates were randomly selected for technical repetitions as a verification of the lack of technical bias during LC-MS/MS process. No significant difference was observed between any repeats. Protein identification was achieved by comparing the MS data to both the UniProt Knowledgebase restricted to *B.* *distachyon* (http://www.uniprot.org/, October 2013) and a contaminant database, including human keratins and trypsin. Enzymatic cleavage was declared as a tryptic digestion with one possible miscleavage event. The fixed modifications of Cys residues by iodoacetamide and the possible oxidation of Met residues were considered. Precursor mass and fragment mass tolerance were set at 5 ppm and 0.5 Da, respectively. The results from the X!Tandem analysis were filtered according to a peptide *e*-value below 0.01 in the X!Tandem pipeline. Proteins were identified using at least two specific peptides.

### 2.4. Functional Annotation

Bioinformatics analysis of the identified proteins was performed using the ProtAnnDB tool (www.polebio.lrsv.upstlse. fr/ProtAnnDB/) [27]. The sub-cellular localization of the proteins was predicted using different software programs: TargetP (http://www.cbs.dtu.dk/services/TargetP/), Predotar (https://urgi.versailles.inra.fr/predotar/predotar.html), SignalP (http://www.cbs.dtu.dk/services/SignalP/), Phobius (http://phobius.sbc.su.se/) and TMHMM (http://www.cbs.dtu.dk/services/TMHMM-2.0/). The selection of the CWPs corresponded to the intersection results obtained with these above software programs. The functional domains or motifs were predicted using PROSITE (http:// prosite.expasy.org/), Pfam (http://pfam.xfam.org/), and InterProScan (http://www.ebi.ac.uk/Tools/pfa/iprscan5/) bioinformatics programs [27]. The proteomic data of the present work have been included in the WallProtDB database (www.polebio.lrsv.ups-tlse.fr/WallProtDB/) [28]. The WallProtDB tools were used for cell wall proteome comparisons given in the discussion section.

### 2.5. Quantification and Statistical Analysis

The spectra with confident identifications were collected for quantification from the X!Tandem pipeline by the MassChroQ tool (version 2.1.0). MassChroQ is available at http://pappso.inra.fr/bioinfo/masschroq and was used for label-free quantification based on eXtracted Ion Chromatograms, usually noted as the XICs. Peptide quantification was performed by integration of extracted ion current in the peak at the expected retention time. As distortions can occur between runs, the MS retention times were aligned to a reference before extracting the ion chromatogram. To perform this alignment, 36 groups of similar runs corresponding to the same gel slices (five replicates) at a given development stage were defined (Supplementary Figure S1). Within each group, the run containing the highest number of identified peptides was selected as the reference. In our study, the XICs built within a 10 ppm range of masses were further normalised as described [29] and expressed as the most intensive peaks.

The resulting table of peptide XICs was then processed as described in Supplementary Figure S1 by the R statistical environment (version 3.1.0). To compare 1D-E lanes, which represent biological replicates for the three stages, a correction factor was applied to the values. This factor was the ratio of the mean XIC-lanes to the current XIC-lane, where a XIC-lane is the sum of XICs along a lane. The abundance value of one peptide was the result of the aggregation of XICs related to identical peptides across the multiple gel slices from the same biological replicate within one stage. Identical peptides shared the same sequence identification and the same *m*/*z* (for both 2+ and 3+ parent ions with aligned RT).

The resulting individual values of the abundance of the peptides were further processed when they were observed in at least two biological replicates and when they were specific to one protein. A relative quantification of the proteins was performed when they were identified by at least two specific peptides and were computed by summing the values of the peptides. Only proteins identified among at least three biological replicates within at least one developmental stage were conserved. In addition, the sub-cellular localization predictions of the resulting list of proteins were considered to select the CWPs to focus on in this study. Statistical tests were performed on log_10_-transformed data. The missing values between biological replicates were replaced by the minimum value of the lane they belonged to. Missing values between stages were not replaced, as the study was conducted separately on proteins shared among the three stages and between two stages.

PCA was performed using the ade4 package (Version 1.16-2, available at http://pbil.univ-lyon.fr/ADE-4) to highlight the variable correlations according to the stages. One-way ANOVAs were used to determine the proteins with a significant developmental stage effect. Their abundances were considered to be significantly different for p-values below 0.001. This dataset of differently abundant proteins was further used with hclust and the gplots package to build heat maps (version 2.14.1, available at https://cran.r-project.org, the Comprehensive R Archive Network). We extrapolated the preferential stages of protein clusters according to their abundance from the three stages heat map and its row flanking dendrogram by fixing the value of 1.1 as threshold within the related distance matrix.

### 2.6. Immunolabelling

After the 1D-E separation, the proteins (10 μg per sample) were electro-transferred to a nitrocellulose membrane (Invitrogen, Carlsbad, CA, USA). The PageRuler Prestain Protein Ladder (SM0671, Thermo Fisher) was used as a mass marker. The presence of proteins was revealed using antibodies against *B. distachyon* GH18 (Bradi4g09430) and GH3 (Bradi1g08570), which have been previously described [15]. These antibodies were used at a 1:2000 dilution, and detection was performed using an alkaline phosphatase-conjugated goat anti-rabbit antibody (1:3000 dilution) as described [4]. Sample preparation for the immunofluorescence labelling of grain sections was conducted as previously described [4].

### 2.7. Semi-Quantitative RT-PCR

The total RNA was extracted from 9, 13 and 19 DAF grains of *B. distachyon* using a RNA kit (Qiagen, Courtaboeuf, France). RNA samples were treated twice with the DNase Set (Qiagen) and then purified using the RNeasy MinElute Cleanup Kit (Qiagen) by following the manufacturer’s instructions. Reverse transcription was carried out with 2 μg of total RNA, random hexamers, and the Transcriptor First Strand cDNA Synthesis Kit (Roche Applied Science, Mannheim, Germany). PCR reactions were performed using KOD polymerase (Novagen Inc., Madison, WI, USA) with the appropriate buffer and the forward and reverse primers (1 μL of 10 μM solutions) and in a total volume of 25 μL. The primers used for the PCR reactions were as follows: RT-GH18s (5′-GCCAGGATGACAAGAAGTGC-3′), RT-GH18r (5′-CCGTAGTTGCTCTGCTTGTC-3′), RT-GH3s (5′-ATCATCGACAAACGTGGGCT-3′), RT-GH3r (5′-TGGTTGGCGTGCATTTTCTG-3′), SamDCs (5′-CGGCAAGCTTGCTAATCTGCTCCAAT-3′) and SamDCr (5′-CAGAGCAACAATAGCCTGGCTGGC-3′). The PCR products were separated on 2% agarose gels by electrophoresis.

## 3. Results

### 3.1. Extraction of Proteins from Cell Walls of the B. distachyon Grain at Three Developmental Stages

**Figure 1:**
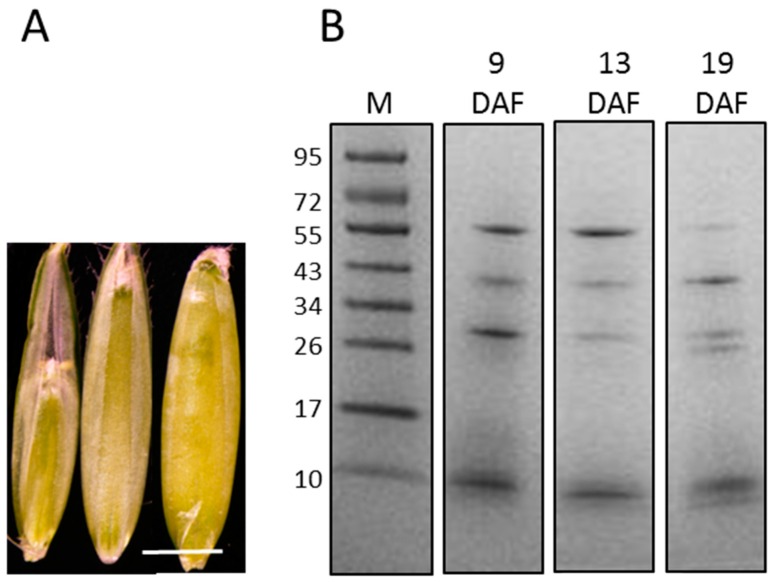
The extraction of proteins from the cell walls of the *B. distachyon* grain at three stages of development. (**A**): *B. distachyon* grains in the palea at 9 DAF (**left**), 13 DAF (**middle**) and 19 DAF (**right**). Scale bar = 2 mm; (**B**) The 1D-E profiles of the proteins extracted from the cell wall of grains harvested at 9, 13 and 19 DAF. Ten μg of total proteins from each sample were loaded on a 12% polyacrylamide gel and stained with Coomassie Brilliant Blue. M: molecular mass markers (kDa).

The three developmental stages that were chosen for this study are representative of distinct steps of the *B. distachyon* grain development. This choice was dependent on both physiological interests and technical constraints. In particular, we chose to work with developmental stages preceding the storage protein accumulation step to prevent a high level of contamination by these proteins. Before 8–9 DAF, harvesting grains would have been too challenging because of their very small size, and would have required too many grains to collect a sufficient amount of material. The three retained developmental stages had morphological and physiological characteristics that cover the main steps of grain development before its maturation and its desiccation. The earlier stage (9 DAF) corresponds to the end of the cellularisation step. At morphological level, the grain fills only a half of the palea (Figure 1A). The endosperm is fully cellularised, but the cells continue to divide. Their cell walls also start to thicken. At 13 DAF, the developing grain reaches its maximum length, but is not filled. The aleurone layers are distinguishable from the endosperm cells. The accumulation of starch in the endosperm has started. At 19 DAF, the grain is filled, and the endosperm cells are well expanded. Storage protein accumulation begins, with the presence of numerous protein vesicles in the endosperm cells [5].

To have an overview of grain development, a proteomic analysis of the three developmental stages of *B. distachyon* grain (9, 13 and 19 DAF) was performed. Briefly, the cell walls were isolated, and the proteins were extracted with CaCl_2_ and LiCl solutions prior to protein separation by 1D-E and LC-MS/MS analyses (Supplementary Figure S1).

From 500 mg of fresh grains, approximately 70 mg of lyophilised cell walls were obtained for each biological replicate. An average of 150 μg of proteins was extracted for each sample. It is interesting to note that the latest the grains were harvested, the highest the amount of lyophilised cell wall was recovered. In contrast, a lower amount of proteins was extracted from the latest developmental stage (19 DAF). The 1D-E protein profiles obtained at 9, 13 and 19 DAF showed major stained bands with apparent molecular masses between 10 and 95 kDa. The electrophoretic profiles appeared more similar at 9 and 13 DAF compared to that at 19 DAF (Figure 1B).

### 3.2. Proteomic Analysis

To account for biological variability, the proteins were extracted from five independent biological replicates per stage to be analysed by LC-MS/MS after 1D-E separation. Only proteins identified with at least two specific peptides present within the same extract were retained. A dataset of 1122 proteins was deduced from this data analysis and considered for further analyses.

Only proteins with a predicted signal peptide that lacked a C-terminus endoplasmic retention signal (KDEL or HDEL) were conserved for further analysis and considered as CWPs. Among the 1122 proteins identified, approximately 40% fulfilled these criteria, leading to a total of 466 CWPs, with all developmental stages taken into account (Supplementary Table S1). A list of these proteins and the corresponding LC-MS/MS data are available online in the WallProtDB database (http://www.polebio.lrsv.ups-tlse.fr/WallProtDB/).

**Figure 2:**
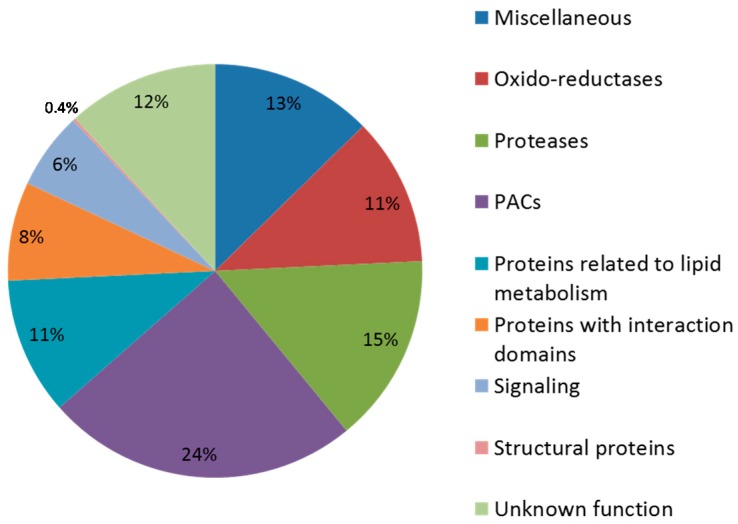
Distribution of CWPs into functional classes according to their predicted functions.

Predictions of the functional domains were performed using the ProtAnnDB tool [27]. CWPs were distributed into nine functional classes according to [30] (Figure 2). The class of proteins that act on cell wall polysaccharides (PACs) was the most represented (24% of CWPs), followed by that of proteases (15% of CWPs) and that of miscellaneous proteins (13%). Twelve percent of CWPs were proteins with unknown functions. Oxido-reductases and proteins related to lipid metabolism were also well represented, each with 11% of the CWPs. Other functional classes represented less than 10% of the CWPs, with the identification of only two structural proteins.

### 3.3. Variations of CWP Abundance during Grain Development

#### 3.3.1. Distribution among the Three Developmental Stages

**Figure 3:**
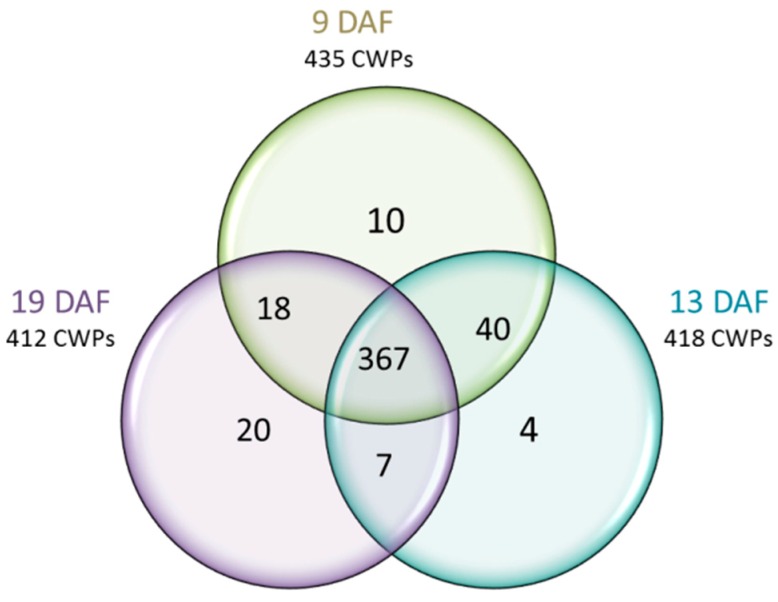
Venn diagram showing the distribution of CWPs according to the three stages of development of the *B. distachyon* grain (see Supplementary Table S1C in which the CWPs identified at one or two developmental stages are listed).

Among the 466 non-redundant CWPs identified, 435, 418 and 412 were found at 9, 13 and 19 DAF, respectively (Figure 3; Supplementary Table S1), with 367 CWPs identified at all three stages. Forty CWPs were found at 9 and 13 DAF, seven at 13 and 19 DAF, and 18 at 9 and 19 DAF. Only 34 CWPs were identified in a single stage (10 at 9 DAF, four at 13 DAF and 20 at 19 DAF). Their relative amounts were found to be close to the median value of the whole protein dataset in these stages, meaning that they were above a potential detection threshold. These CWPs belonged to different functional classes, such as proteases, miscellaneous proteins, PACs or proteins related to lipid metabolism (Supplementary Table S1). When focusing on the number of CWPs in a given functional class, there were only few differences according to the stage (Supplementary Table S2).

#### 3.3.2. Quantitative Analysis of CWPs

**Figure 4:**
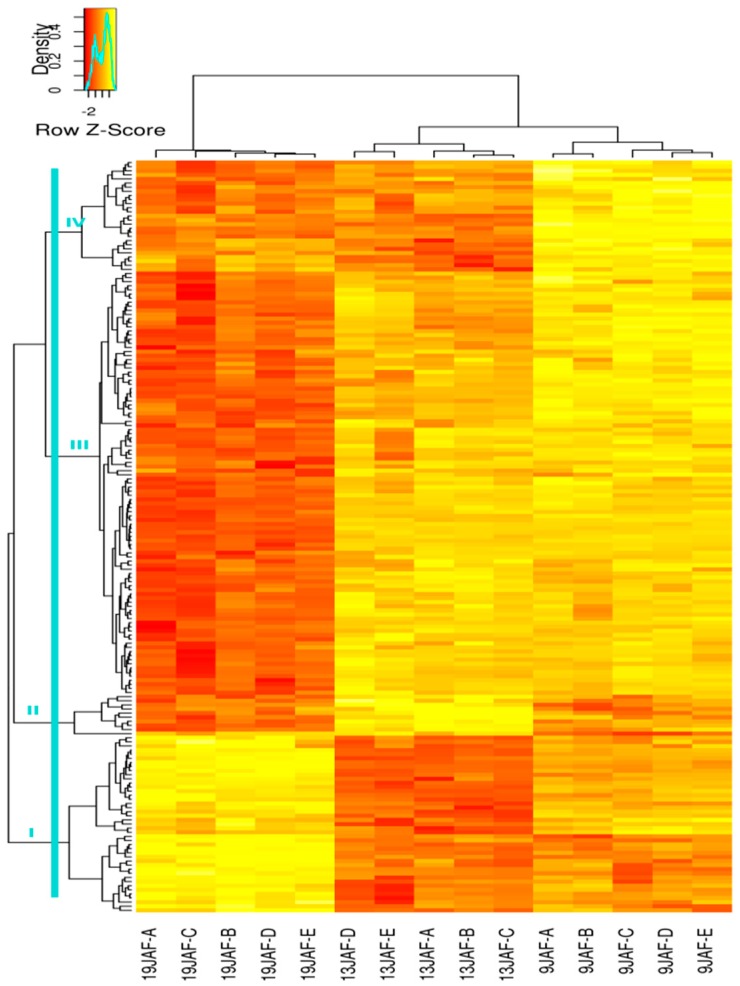
The heat map of the CWPs identified at the three developmental stages of the *B. distachyon* grain. The 5 biological replicates are represented for each stage. The variation in the protein abundance is shown by a colour code ranging from yellow (the most abundant proteins) to red (the less abundant proteins). The blue line represents the extraction level of the clusters. Cluster numbers (from I to IV) are indicated on the heat map.

Most CWPs were found at all three developmental stages of the *B. distachyon* grain (367 CWPs), and 65 CWPs were identified at two of these stages. A quantitative analysis was performed to parse out the variation in the abundance of CWPs identified at several developmental stages. The principal component analysis (PCA) indicated a strong clustering of biological replicates for each of the developmental stages and clearly separated the 19 DAF stage from the others on the first principal component PC1 whereas PC2 separates the two younger stages (Supplementary Figure S2). For the three stages, the median value of the relative amounts of CWPs expressed as log_10_(XIC), was approximately of six (Supplementary Figure S3). The amounts of CWPs identified at a single stage were found close to this median value, indicating their significant presence compared to the other stages. The amount was assessed by an ANOVA indicating significance, and it revealed 195 CWPs for which the amount significantly varied according to the developmental stage. This corresponded to approximately half of the CWPs common to two or three stages. The corresponding heat maps grouped proteins according to their covariance (see Figure 4 for the three stage-comparison and Supplementary Figure S4 for the two stage-comparisons). The five biological replicates per stage were appreciably in the same range for a given CWP, indicating a good precision for the samples. The heat map corresponding to the CWPs expressed at the three developmental stages revealed a large majority of CWPs with a low variation of protein abundance between 9 and 13 DAF compared to the 19 DAF stage and confirmed the trend deduced from the PCA.

Four clusters were extrapolated from the flanking dendrogram of the heatmap to assign CWPs to the developmental stage at which they appeared the most abundant. The larger cluster included CWPs showing a higher abundance at 9 and 13 DAF (Figure 4, cluster number III). Looking at the variation of the amount of proteins, the 13 DAF stage appeared as an intermediate stage between 9 and 19 DAF stages. The functional repartition of the CWPs among clusters reveals that at least four of the nine CWP functional classes (miscellaneous, oxido-reductases, proteases and signaling proteins), are mainly more abundant at earlier stages (Supplementary Table S1). PAC was the functional class with the most highly abundant proteins at the three developmental stages. Among the 157 CWPs that were highly abundant at 13 DAF, class III Prxs were well represented. The lipid transfer proteins (LTPs) were clearly more abundant at 19 DAF than at 9 and 13 DAF.

### 3.4. Focus on Proteins Acting on Cell Wall Polysaccharides (PACs)

PACs were the best represented in our proteomic work and gathered almost one quarter of the CWPs (114, Figure 2). This class of proteins was predominantly represented by GHs (80.7% of PACs), with 92 proteins identified and distributed over 20 GH families (Supplementary Table S1). It also contained 12 expansins, five CEs, four PNGases A and one polysaccharide lyase (PL). The most populated GH families were GH17 and GH28, with 14 and 10 members, respectively.

#### 3.4.1. Repartition of PACs According to the Grain Developmental Stages

**Figure 5:**
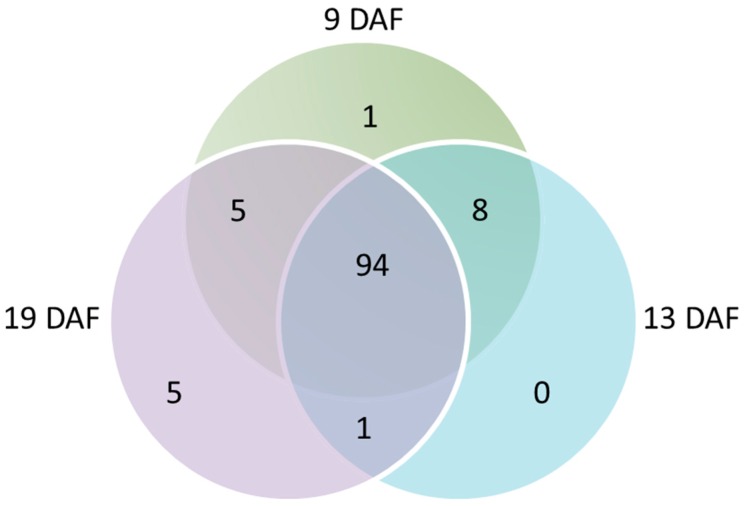
Venn diagram showing the distribution of PACs among the three developmental stages of the *B. distachyon* grain.

A Venn diagram was used to represent the repartition of the PACs at the three studied stages (Figure 5). Almost the same number of PACs was identified for each of them (108 CWPs at 9 DAF, 103 at 13 DAF and 105 at 19 DAF), with most of them shared among the three stages (94 CWPs, i.e., 82.4% of PACs) (Figure 5). Only a few CWPs were found at a single stage (one and five proteins at 9 and 19 DAF, respectively) (Figure 5). They were all GHs, and they belonged to five families: (i) GH32 at 9 DAF and (ii) GH1, 16, 17 and 35 at 19 DAF. Additionally, eight PACs were found at both 9 and 13 DAF (two expansins and six GHs), one CE13 (pectin acylesterase) at 13 and 19 DAF, and five GHs at 9 and 19 DAF (Supplementary Table S1).

#### 3.4.2. Quantitative Analysis of the PACs

Because most PACs were expressed at all three grain developmental stages, the variation in their amount was statistically analysed. Except at 9 DAF, where the proteases were present in the highest amount, PACs were generally the most abundant proteins at all three stages (Supplementary Table S1). As shown above, this class predominantly included GHs but also expansins, CEs, PNGases and PL. The heat map representing the covariance of the PAC abundance is shown in Supplementary Figure S5. It had a pattern of covariance quite similar to that representing all the CWPs (see Figure 4), and clearly indicated the best covariance between 9 and 13 DAF. According to the developmental stage, some PAC families were distinguishable by their abundance levels. At the early developmental stages, 9 and 13 DAF, PACs were present in high amounts, as well as numerous expansins. The GH28 family was well represented, with two members identified only at these two early stages (Supplementary Table S1). Among the PACs that showed high abundance at 19 DAF, the GH51 family stood out, with the higher amount of all three members identified in this work.

#### 3.4.3. Experimental Validation of the Proteomic Quantitative Analysis

**Figure 6:**
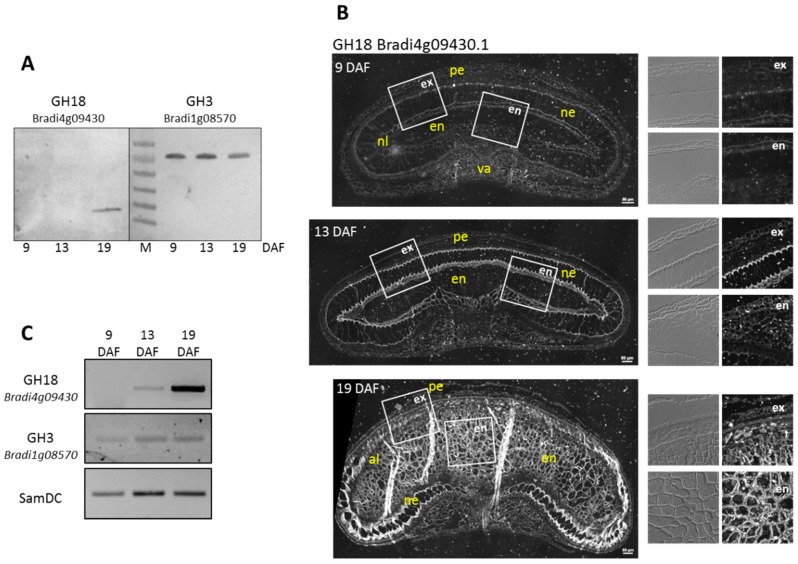
The abundance profiles of two GHs during *B. distachyon* grain development and variations in the corresponding transcript levels. (**A**) Immunoblotting performed with anti-GH18 (Bradi4g09430) and anti-GH3 (Bradi1g08570) antibodies. The proteins were extracted from *B. distachyon* cell wall grains harvested at 9, 13 and 19 DAF. GH3 (Bradi1g08570): theoretical molecular mass: 67.5 kDa; apparent molecular mass: 80 kDa; GH18 (Bradi4g09430): theoretical molecular mass: 30.6 kDa; apparent molecular mass: 28 kDa. (M): protein mass marker; (**B**) Immunolocalization of Bradi4g09430 in *B. distachyon* grains harvested at 9, 13 and 19 DAF. For each cross section, higher magnifications of the endosperm (white frames “en”) and the external layer region (white frames “ex”) are shown. en, endosperm; pe, pericarp; va, vascular tissue; al, aleurone; ne, nucellar epidermis; nl, nucellar lysate. Scale bars = 50 μm; (**C**) Transcript accumulation profiles of *Bradi4g09430* and *Bradi1g08570* by semi-quantitative RT-PCR using the total RNAs extracted from grains harvested at 9 DAF, 13 DAF and 19 DAF. The *S*-adenosylmethionine decarboxylase gene (*SamDC*) was used for the normalization of the PCR reactions.

To strengthen the significance of our quantitative analysis, we looked (i) at the level of the abundance of two GHs by Western blot analysis and immunocytochemistry and (ii) at the level of accumulation of the corresponding transcripts by semi-quantitative RT-PCR. One GH was a GH18 encoded by *Bradi4g09430* with a high amount at 19 DAF (Supplementary Table S1), and the other was a GH3 encoded by *Bradi1g08570* for which no significant difference in the amount was detected. For Bradi4g09430, both the Western blot analysis and the immunolabelling of the *B. distachyon* grain sections indicated a higher amount of protein at 19 DAF (Figure 6A,B). The *Bradi4g09430* mRNA level was found to increase from 9 to 19 DAF (Figure 6C). Using the same techniques, we validated the absence of variation in the level of abundance of the Bradi1g08570 protein and of the *Bradi1g08570* transcripts (Figure 6A,C).

## 4. Discussion

This proteomics work has allowed the identification of numerous CWPs from the grain of *B. distachyon* at three different developmental stages. Many of them presented a significant variation in their level of abundance according to the stage of grain development, and these variations provided information on the dynamics of cell wall remodelling events. This work has led to significant enlargement of knowledge of the cell wall proteome of *B. distachyon*; among the 466 CWPs identified in this analysis, 111 were newly found. They are distributed into nine functional classes (Supplementary Table S1D). Among them, we found numerous proteins potentially involved in the assembly and remodelling of the grain cell wall. The remaining CWPs were already identified in the cell wall proteomes of grains, culms, leaves and seedlings of *B. distachyon* [15,16,17]. Today, the global cell wall proteome of *B. distachyon* contains 585 CWPs, with 253 CWPs specifically found in the cell wall proteome of grain (data from this analysis and [16]), and 233 found both in the grain and in vegetative organs (Supplementary Figure S6) [15,17]. To our knowledge, this is the largest cell wall proteome from a monocot plant species, not far behind that of the dicot model plant *A. thaliana* which comprises 710 CWPs according to the WallProtDB database.

An increasing amount of lyophilised cell wall material was obtained during the time course of the development of grains, i.e., from 9 to 19 DAF. This is consistent with the thickening of cell walls during grain development, most probably in relation to the accumulation of mixed-linked β-glucan that acts as both a storage component and a structural molecule. Inversely, larger amounts of proteins were extracted from the grain at earlier stages of development. This could be explained by a better extractability of the proteins due to a looser polysaccharide network at these stages. In addition, the higher metabolic activities during cell growth could explain a larger amount of proteins at the early developmental stage. The quantitative analysis showed that many CWPs were more abundant at 9 and 13 DAF, especially proteases that have a critical role in defence reactions [31] and PACs, with a particularly high abundance of GHs and expansins known to play key roles in cell growth [18].

The quantitative proteomic analysis revealed clear differences between the stages. In particular, a large difference was observed between the cell wall proteomes of grains harvested at 19 DAF and those harvested at 9 and 13 DAF, with an increased number of proteins present in higher amounts at the early stages in almost all functional classes. This is consistent with strong cell expansion activity up to 13 DAF, requiring numerous CWPs specialised in cell wall deposition. The 13 DAF stage appeared to be intermediate between the 9 and 19 DAF stages, with less highly abundant CWPs than at 9 DAF and more than at 19 DAF. It is interesting to note that 19 DAF is the developmental stage that accounted for the most CWPs identified at a single stage whereas few of CWPs shared between three stages were highly abundant at this stage. Altogether, the quantitative analysis highlighted the critical differences between the cell wall proteomes at the different stages of grain development, thus demonstrating the importance of the regulation of the level of CWP amount in the dynamics of cell growth and cell wall deposition and remodelling. The significance of our findings has been further investigated with two GHs for which we could show that their gene expression was regulated at the transcript level.

Altogether, the CWP functional class that was best represented at the three developmental stages of the *B. distachyon* grain was that of PACs. It represented almost a quarter of the total number of CWPs, a proportion which has been found in the cell wall proteomes of various organs of *B. distachyon*, *A. thaliana* and *O. sativa* [15,32,33,34,35]. GHs, expansins, CEs and PNGases were the most represented protein families in the cell wall proteomes of the *B. distachyon* grain. These CWPs are known to participate in the remodelling of cell wall polysaccharides, but little is known about the precise mechanisms they contribute to [36,37]. The availability of information concerning their changes in abundance during grain development will be critical to better understanding of their roles in grains.

The members of some GH families were highly abundant preferentially at one of the three developmental stages of the grain, with members only identified at one stage. A contrasted situation was observed in the GH17 family. Although 10 out of its 14 members showed no difference in abundance between the three developmental stages, two of them were only found at 19 DAF (discussed below), and two others were highly abundant at early stages of development. The GH1 family was also well represented with nine members identified, which were mainly abundant at 9 and 19 DAF except one, which was identified only at 19 DAF. It was proposed that GH1 and GH17 β-glucosidases could participate in the turn-over of mixed-linked β-glucans. These polysaccharides are deposited during the cellularisation phase and remain present throughout the development of the endosperm, which is consistent with the presence of the GH1 and GH17 proteins throughout grain development [38,39]. Regarding the GH32 family, one member was expressed only at 9 DAF (Bradi5g25270.1). Among the three other members identified, one was more abundant at 9 and 13 DAF (Bradi2g61830.1). Numerous activities were assigned to GH32, such as acid cell wall invertase activity. They have also been associated to fructan metabolism, including fructosyltransferase and fructo-exohydrolase activities [40]. In rice, four of the nine genes encoding cell wall invertases have been observed to be expressed in immature seeds and were proposed to play a critical role in carbohydrate supply for developing caryopsis during the early developmental stages of the rice filling phase. It was shown that these cell wall invertases exhibited very different spatial expression patterns in the rice grain [41]. The invertases identified at early developmental stage of the *B. distachyon* grain could also contribute to the growth of the grain.

Although identified at all three studied developmental stages, GH28 were more abundant at 9 and 13 DAF. The identification of numerous proteins in this family was a surprising result because they are known to have polygalacturonase activity (http://www.cazy.org/GH28.html), whereas the grass primary cell walls contain low levels of pectins, contrarily to those of dicots [42]. Polygalacturonases catalyse the degradation of homogalacturonan (HG), an important component of the pectin skeleton in plant cell walls. This degradation has been described to be a crucial event for cell wall elongation during the growth and development of *A. thaliana* seedlings [43]. Recently, pectins (HG and rhamnogalacturonan) have been detected not only in the endosperm and the outer layers of wheat grains but also in the seed coat beneath a thick cuticle [44]. Pectins were also detected by immunolabelling the *B. distachyon* grain at maturation, but no information is available concerning the occurrence of pectins at earlier developmental stages [4]. The higher amount of GH28 at 9 and 13 DAF is consistent with the remodelling of early synthesised pectins in the developing grain [44,45]. In addition, only methylated HG was detected at early stages of development of the wheat grain. It was suggested that the methylation of HG promotes the elasticity of the cell wall in expanding cells [46]. At late developmental stages, it was hypothesised that the occurrence of unmethylated HG, resulting in a decrease of wall extensibility, was due to the action of PMEs. Although the degree of methylated HG has to be confirmed in the *B. distachyon* grain, the hypothesis of the demethylesterification of HG during grain development could be strengthened by the identification of two PMEs of the CE8 family. Although they are present at the three developmental stages, these enzymes seemed to be more abundant at 19 DAF, when the cells are expanding. In addition, the presence of members of the CE13 family at the three grain developmental stages could be related to the deacetylation of pectin during grain development, thus affecting cell extensibility by changing the physicochemical properties of the cell wall polysaccharides [47].

Most of the numerous expansins identified in our proteomic study were preferentially abundant at both early developmental stages. Expansins are secreted to the cell wall and have the ability to loosen plant cell walls in a non-enzymatic but pH-dependent manner, thus allowing cell expansion [48]. It was demonstrated in wheat that expansins play a major role in crop yield by affecting grain development and size [49]. In this case, expansins were notably abundant in the pericarp during early grain expansion, and subsequently, in both the endosperm and pericarp. Like expansins, many class III Prxs were identified, and they were mainly abundant at 9 and 13 DAF. They represent a large part of the oxido-reductase functional class. They are known to participate in cell wall dynamics by contributing to either loosening or stiffening of the cell wall components [37,50]. It was recently showed that the over-expression of a wheat expansin in tobacco plants strongly enhanced the peroxidase activity of a cell wall bound fraction, thus suggesting that there may be some relationship between class III Prxs and expansins during plant cell growth and in response to environmental stresses [51].

According to the clusters extracted from the heat map of the whole CWPs identified at the three developmental stages of the *B. distachyon* grain, the only protein family that appeared to be more highly abundant at 19 DAF compared to 9 and 13 DAF was the LTP family, with three members only identified at this stage. LTPs are abundant proteins in plant seeds. The localization of LTPs at the cell wall has been demonstrated in several plants, such as *A. thaliana*, *Vigna unguiculata* seeds or *Brassica oleracea* [52]. In addition to their potential roles in antimicrobial defence and defence signalling, LTPs have been proposed to act in cuticle deposition and cell wall remodelling [53]. While not directly demonstrated, LTPs have been proposed to be carriers of the lipid precursors of the cuticle or structural components of the cuticle. In wheat, a thick cuticle is deposited on the outer surface of the seed coat [44]. Two other thinner cuticles are formed in the outermost layer of the pericarp and outside the nucellar epidermis. These cuticles seem to form at a relatively early stage of grain development: this could explain the abundance of LTPs at 19 DAF which corresponds to the beginning of the *B. distachyon* grain filling.

Five GH members were identified only at 19 DAF. Among them, we found one member of the GH1 family that has mainly β-glucosidase activity, one member of the GH35 family with β-galactosidase activity that modifies cell wall components, including pectins and arabinogalactan proteins, two members of the GH17 family that are mainly glucan endo-1,3-β-glucosidases, and one member of the GH16 family which contains numerous xyloglucan endotransglycosylase/hydrolases (XTHs). These latter proteins are known to cleave xyloglucan (XG) to incorporate new oligoxyloglucans and catalyse the formation of linkages between XG and cellulose [54]. Whereas no or few XGs were detected in the cell wall of *Pooideae*, the presence of XTHs in grasses could be explained by the activity of these enzymes on the more abundant matrix polysaccharides xylans and β-glucans [55,56]. Although these five GHs were specifically identified at 19 DAF, where they could play a critical role, the protein families to whom they belong are not specific to this developmental stage.

Finally, the GH51 family was found to be characteristic of the 19 DAF stage with all members highly abundant at this developmental stage. Moreover, no GH51 has been previously identified in other organs of *B. distachyon* [15,17]. This protein family includes l-arabinofuranosidases and xylosidases. Its involvement in the modification of the heteroxylan fine structure by removing arabinofuranosyl residues from arabinoxylans (AX) during growth and development has been demonstrated in barley [57]. AX is one of the major polysaccharides in grass cell walls and is synthesised relatively late during the development of the grain [38]. In the wheat endosperm, the level of arabinose substitution decreases during the grain filling period, leading to a modification of the physicochemical properties of the cell wall [9]. The higher amount of GH51 at 19 DAF may be related to the debranching of AXs in the *B. distachyon* grain. Biochemical investigations should be performed on the AXs in the *B. distachyon* grain to test this hypothesis.

## 5. Conclusions

Our quantitative proteomic work has allowed not only the identification of numerous CWPs at three critical stages of the *B. distachyon* grain development, but also the importance of the variations of their abundance between 9 and 19 DAF. It provides important knowledge for better understanding of the dynamics of the cell wall during grain development and stresses the importance of CWPs, such as XTHs and polygalacturonases (GH28), during this physiological process. This finding was not expected because both XG and pectins are not abundant polysaccharides in grass cell walls. Deeper investigations need to be conducted to characterize the fine structure of the cell wall polysaccharides during the *B. distachyon* grain development to better correlate the observed proteome changes and the polysaccharide remodelling. Furthermore, the distribution of CWPs in the different tissues of the grain should be considered to determine their precise role in the albumen and the outer layers of the grain during its development. Additionally, the fine analysis of genetically modified plants affected in the expression of candidate genes identified in this study should allow the understanding of their role at specific developmental stages of the *B. distachyon* grain.

## Supplementary Materials

The following are available online at www.mdpi.com/2227-7382/4/3/21/s1, Table S1: (**A**) Proteins extracted from the cell walls of the *B. distachyon* grain at the three developmental stages that are predicted to be secreted (called CWPs); (**B**). Statistical analysis performed on the data corresponding to *B. distachyon* CWPs identified at two or three grain developmental stages; (**C**). *B. distachyon* CWPs identified only at one or two grain developmental stages; (**D**). *B. distachyon* CWPs newly identified, Table S2: The distribution of CWPs into functional classes at the three developmental stages of the *B. distachyon* grain according to their predicted function, Figure S1: The workflow of our quantitative proteomic approach, Figure S2: Principal Component Analysis (PCA) performed using the relative amounts of the CWPs at the three developmental stages of the *B. distachyon* grain, Figure S3: The distribution of log-transformed values of CWPs relative amounts, Figure S4: Heat maps of relative amounts of the CWPs identified at only two grain developmental stages, Figure S5: The heat map of the PACs identified at the three developmental stages of the *B. distachyon* grain, Figure S6: Venn diagram showing the distribution of CWPs among the grains and the others tissues/organs of *B. distachyon.*
