# Supplementary material for: Understanding the Remodelling of Cell Walls during Brachypodium distachyon Grain Development through a Sub-Cellular Quantitative Proteomic Approach
Source: Proteomes. 2016 Jun 24;4(3):21. doi: 10.3390/proteomes4030021 (PMC5217356; doi:10.3390/proteomes4030021)
Supplement: Supplementary file 1 [file proteomes-04-00021-s001.zip › proteomes-04-00021-supplementary/proteomes-04-00021-supplementary.pdf]

Supplementary Materials: Understanding the Remodelling of Cell Walls during *Brachypodium distachyon* Grain Development through a Sub-Cellular Quantitative Proteomic Approach

Mathilde Francin-Allami, Virginie Lollier, Marija Pavlovic, Hélène San Clemente, Hélène Rogniaux, Elisabeth Jamet, Fabienne Guillon and Colette Larré

Table S2. The distribution of CWPs into functional classes at the three developmental stages of the *B. distachyon* grain according to their predicted function.

Functional Classes	9 DAF	13 DAF	19 DAF
Miscellaneous	55	55	48
Oxido-reductases	51	52	47
Proteases	67	62	59
Proteins acting on cell wall polysaccharides	108	103	105
Proteins related to lipid metabolism	46	42	49
Proteins with interaction domains	32	31	28
Signaling	28	26	24
Structural proteins	2	2	2
Unknown function	46	45	50

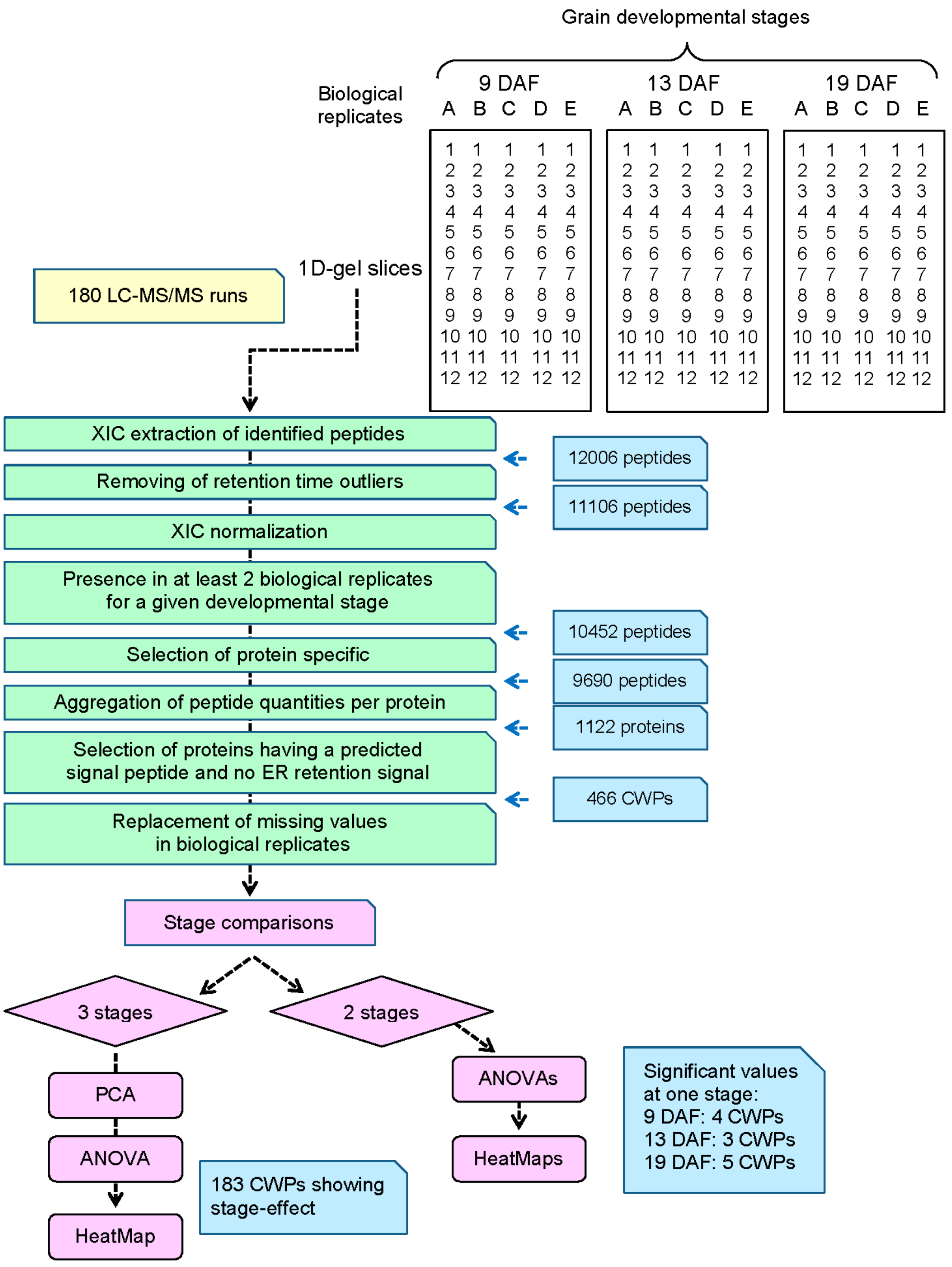

Figure S1. The workflow of our quantitative proteomic approach.

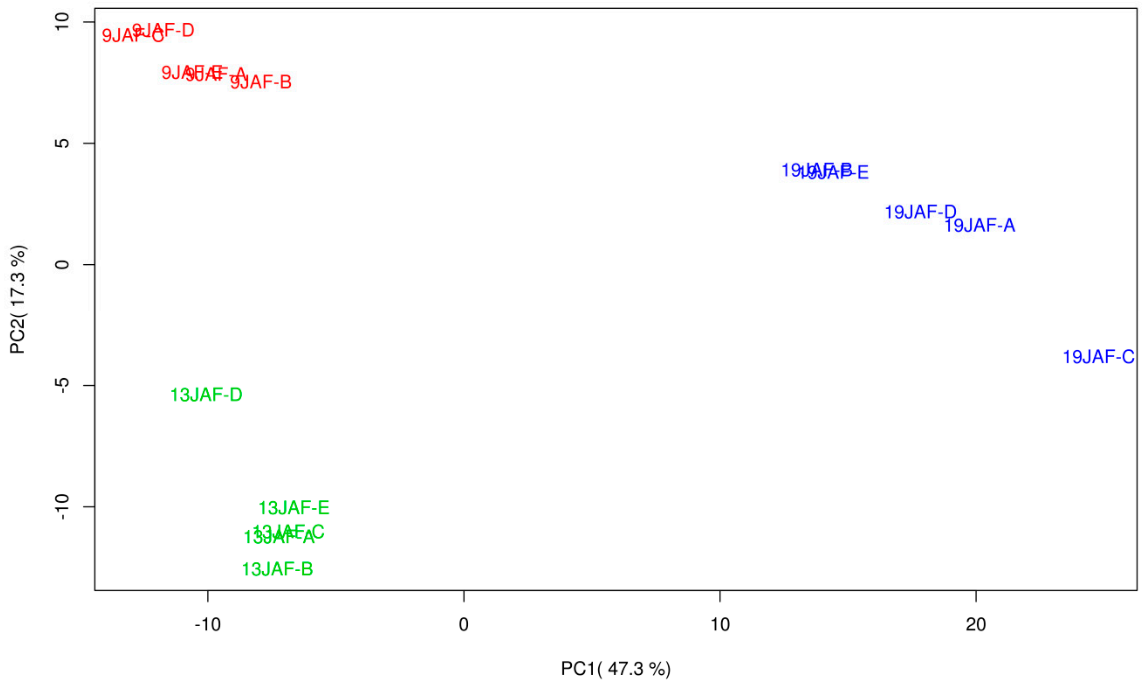

Figure S2. Principal Component Analysis (PCA) performed using the relative amounts of the CWPs at the three developmental stages of the *B. distachyon* grain. Individual map of biological replicates (**red**: 9DAF samples, **green**: 13DAF, **blue**: 19DAF samples) along principal components 1 and 2.

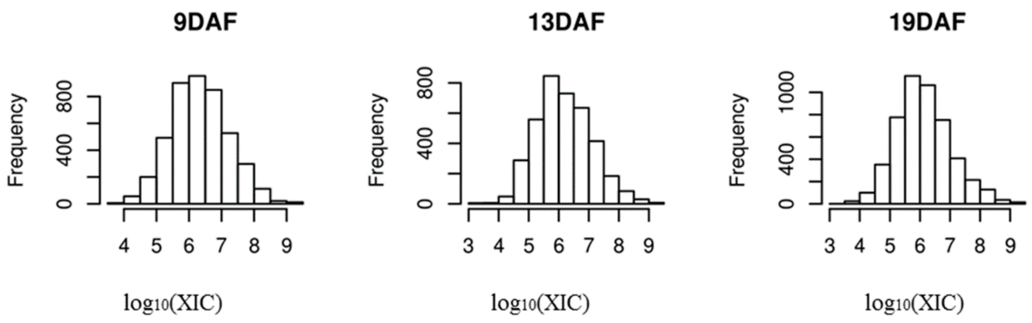

Figure S3. The distribution of log10-transformed values of CWPs relative amounts (that is log10(XIC), where XIC of a CWP is the sum of peptide normalised XICs identifying the CWP) for the three developmental stages.

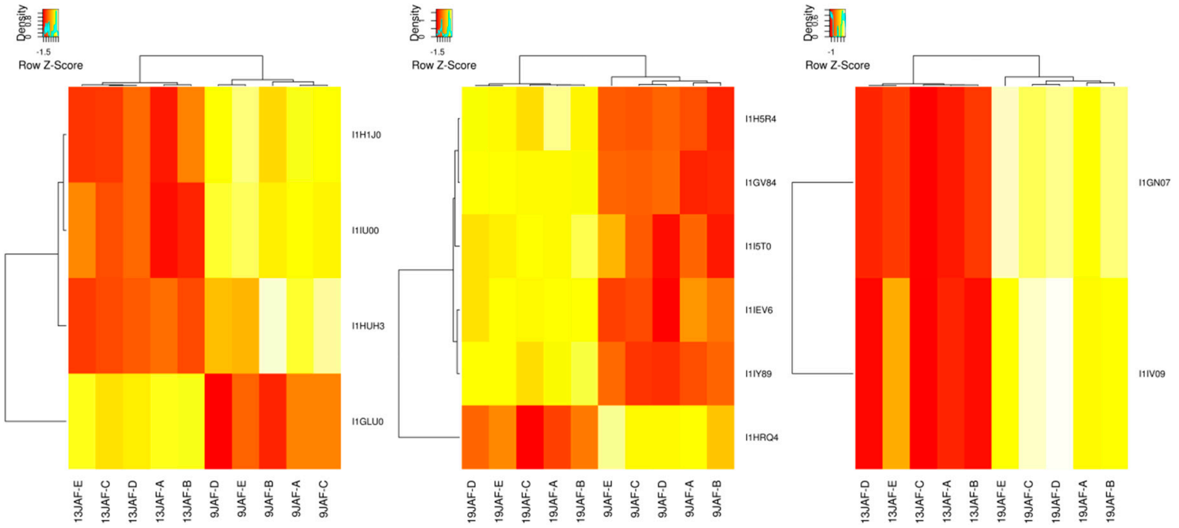

Figure S4. Heat maps of relative amounts of the CWPs identified at only two grain developmental stages. The variation in the protein abundance is shown by a color code ranging from **yellow** (proteins the most accumulated) to **red** (for proteins the less accumulated).

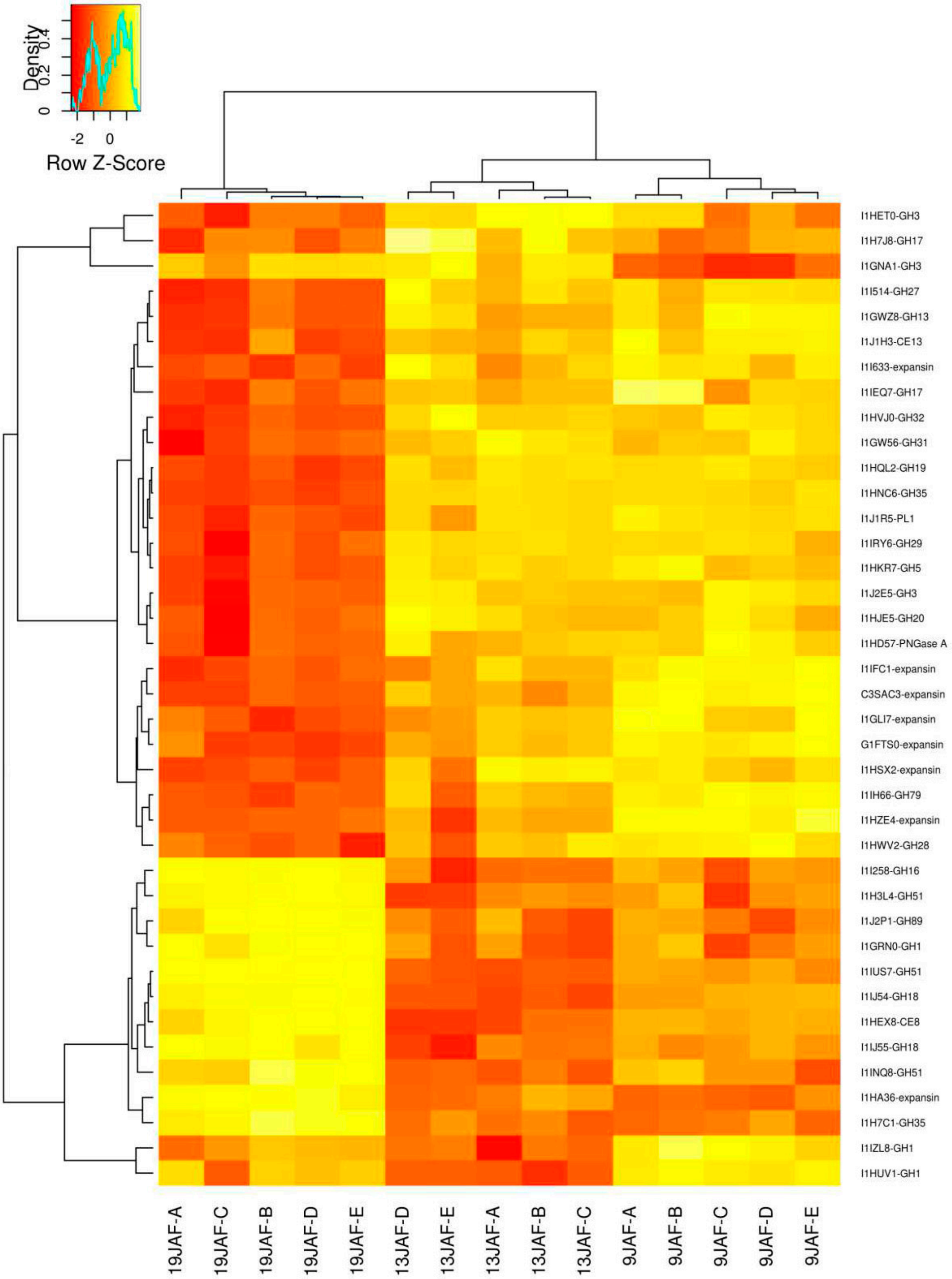

Figure S5. The heat map of the PACs identified at the three developmental stages of the *B. distachyon* grain. The 5 biological replicates are represented for each stage. The variation in the protein abundance is shown by a colour code ranging from **yellow** (the most abundant proteins) to **red** (the less abundant proteins).

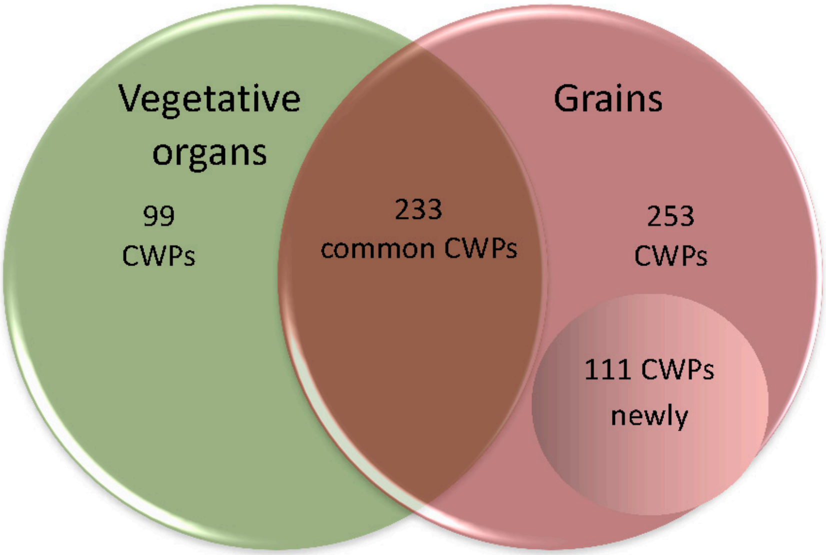

Figure S6. Venn diagram showing the distribution of CWPs among the grains and the others tissues/organs of *Brachypodium distachyon* (according to the data listed in the WallProtDB database).
